# Detection and characterization of bovine hepacivirus in cattle and sheep from Hulunbuir, northeastern China

**DOI:** 10.3389/fcimb.2025.1540849

**Published:** 2025-01-28

**Authors:** Jingge Ma, Zhiwei Wei, Liang Li, Wei Wang, Ziyan Liu, Ning Liu, Feng Wei, Xiangyu Zheng, Zedong Wang

**Affiliations:** ^1^ Department of Infectious Diseases, Center of Infectious Diseases and Pathogen Biology, Key Laboratory of Organ Regeneration and Transplantation of The Ministry of Education, The First Hospital of Jilin University, Changchun, Jilin, China; ^2^ Laboratory of Pathogen Microbiology and Immunology, College of Life Science, Jilin Agricultural University, Changchun, Jilin, China; ^3^ Department of Neurology and Neuroscience Center, The First Hospital of Jilin University, Changchun, Jilin, China; ^4^ Changchun Veterinary Research Institute, Chinese Academy of Agricultural Sciences, Changchun, Jilin, China; ^5^ Hulunbuir Animal Disease Control Center, Hailar, Inner Mongolia Autonomous Region, China

**Keywords:** bovine hepacivirus (BovHepV), sheep, cattle, phylogenetic evolution, China

## Abstract

**Background:**

Bovine hepacivirus (BovHepV) is a recently identified member of the expanding genus *Hepacivirus* within the family *Flaviviridae*. However, the genetic diversity, geographical distribution, and host range of the virus remains poorly understood.

**Methods:**

In this study, serum samples from cattle and sheep were obtained in Hulunbuir and pooled to establish RNA libraries, which were then analyzed using transcriptome sequencing. BovHepV-positive samples were confirmed using semi-nested PCR with primers designed based on the obtained viral sequences. Comprehensive bioinformatics analyses were employed to assess sequence similarity, phylogenetic evolution, and recombination of the obtained viral strains.

**Results:**

A total of 988 serum samples from sheep (520) and cattle (468) were collected from 12 administrative districts in Hulunbuir from June to August, 2022. Semi-nested PCR revealed 6 BovHepV-positive districts with prevalence ranging from 2.0% to 35.0% in cattle, and one BovHepV-positive district with prevalence of 2.5% in sheep. The nucleotide sequence identities between viral strains from sheep and cattle ranged from 91.3% to 93.8%, while the amino acid sequence identities were between 95.4% and 96.7%. Phylogenetic analyses classified the obtained BovHepV strains within subtype G, genotype 1. Recombination analysis revealed the intergenerational relationships among the viral strains obtained from cattle and sheep.

**Conclusion:**

We identified genetic diversity in subtype G strains in cattle and detected a BovHepV strain in a sheep for the first time in northeastern China, confirming cross-species transmission and co-circulation between cattle and sheep, thus expanding the virus’s host range.

## 1 Introduction

Bovine hepacivirus (BovHepV), classified under the genus *Hepacivirus* in the *Flaviviridae* family, was initially detected in serum samples from cattle in Germany and Ghana in 2015, and has since been found to be widely distributed across seven continents (Baechlein et al., 2015; Corman et al., 2015; Canal et al., 2017; Sadeghi et al., 2017; Lu et al., 2018; Yeşilbağ et al., 2018; Lu et al., 2019; Elia et al., 2020; Breitfeld et al., 2022). BovHepV exhibits hepatotropic properties and is capable of inducing both chronic and acute infections in cattle (Baechlein et al., 2015; Corman et al., 2015; Baechlein et al., 2019). However, the true clinical significance and public health risk associated with the virus remain unclear. BovHepV contains a single-stranded positive-sense RNA genome that is approximately 8.8 kb in length, encoding a polyprotein which can be processed into three structural proteins (Core, E1, and E2) and seven non-structural proteins (p7, NS2, NS3, NS4a, NS4b, NS5a, and NS5b) by proteases of both host and viral (Baechlein et al., 2015; Corman et al., 2015). As the sole member of the Hepacivirus N species, BovHepV strains are categorized into two genotypes, with genotype 1 further divided into eleven possible subtypes from A to K (Smith et al., 2016; Shao et al., 2021; Breitfeld et al., 2022).

In China, BovHepV has been identified in cattle populations spanning a broad geographic range, encompassing at least nine provinces: Guangdong, Chongqing, Sichuan, Jiangsu, Yunnan, Henan, Inner Mongolia, Shandong, and Heilongjiang (Deng et al., 2018; Lu et al., 2018; Qiang et al., 2020; Liu et al., 2022a; Lu et al., 2022; Yuan et al., 2023). Additionally, BovHepV has been detected in blood-feeding ticks obtained from cattle in the provinces of Heilongjiang and Guangdong (Shao et al., 2021; Yuan et al., 2023). To date, all strains identified in provinces other than Yunnan and Guangdong have been classified as subtype G within genotype 1 (Lu et al., 2018; Qiang et al., 2020). Hulunbuir, located in Inner Mongolia, northeastern China, is recognized for its extensive sheep and cattle husbandry (Li et al., 2017). Our previous study has identified two BovHepV subtype G strains in this area (Liu et al., 2022a). Nevertheless, the genetical diversity, geographical distribution, and cross-species transmission of the virus are still not well understood. Given cross-species transmission events of BovHepV in cloven-hoofed animals like red deer in the Czech Republic and wild boars in Italy (Smith et al., 2016; Breitfeld et al., 2022; de Martinis et al., 2022), and considering the communal and extensive grazing practices for cattle and sheep in Hulunbuir, we performed transcriptome sequencing on serum samples from both species to investigate the virus’s genetic diversity and circulation in this region.

## 2 Materials and methods

### 2.1 Ethics statement

The animal studies were approved by Animal Administration and Ethics Committee of Changchun Veterinary Research Institute of the Chinese Academy of Agricultural Sciences. The studies were conducted in accordance with the local legislation and institutional requirements. Written informed consent was obtained from the owners for the participation of their animals in this study.

### 2.2 Sample collection

Blood samples were collected from 12 administrative regions in Hulunbuir, northeastern China, between June and August 2022. Blood collection needles and blood vessels are disposable to prevent reuse. Serum was separated from the blood of both cattle and sheep through centrifugation at 500 rpm for 10 minutes and subsequently stored at −80°C until use.

### 2.3 RNA sequencing and transcriptome analysis

Serum samples (20 μL each) from cattle or sheep were pooled in groups of 60-80 based on species and collection sites to construct RNA libraries, as previously described (Liu et al., 2022a). In brief, after digested with micrococcal nuclease (NEB, USA) at a temperature of 37°C for two hours, the serum samples were extracted for RNA using the TIANamp Virus RNA Kit (TIANGEN, China). RNA sequencing was performed at Tanpu Biological Technology in Shanghai, China. RNA from each pool was prepared for library construction using the NEBNext Ultra RNA Library Prep Kit (NEB, USA) following the manufacturer’s instructions and sequenced on an Illumina NovaSeq 6000 System. Each library was sequenced separately to prevent cross-contamination.

The raw sequencing data were processed according to the previously described methodology (Ren et al., 2021). First, low-quality reads, ribosomal RNA, host contaminants, and bacterial sequences were removed using the BBMap tool (https://github.com/BioInfoTools/bbmap). After that, the remaining reads were assembled into contigs utilizing SPAdes v3.14.1 (https://github.com/ablab/spades) and SOAPdenovo v2.04 (https://github.com/aquaskyline/SOAPdenovo-Trans) (Xie et al., 2014; Prjibelski et al., 2020). Following the mapping of sequences against the non-redundant nucleotide (nt) and protein (nr) databases sourced from GenBank using BLAST+ v2.10.0, host and bacterial sequences were removed. The identification of viruses was performed by remapping the reads to the assembled contigs with Bowtie2 v2.3.3.1.

### 2.4 BovHepV detection and complete genome amplification

Viral RNA was extracted from serum samples utilizing the TIANamp Virus RNA kit (TIANGEN, China), and then reverse transcripted into cDNA using the PrimeScript 1st Strand cDNA Synthesis kit (TaKaRa, Japan) following the manufacturer’s instructions. Subsequently, semi-nested PCR was performed to detect BovHepV RNA in the serum samples of both cattle and sheep, using primers designed based on the BovHepV sequences derived from RNA sequencing (**Supplementary Table 1**). To prevent cross-contamination, all steps were performed in separate rooms conducted with nucleic acid scavengers and UV irradiation. In each PCR reaction, three negative controls containing sterile water instead of cDNA template were included. BovHepV positive samples with high genetic diversity were amplified for complete genome using the over-lapping primers designed based on the subtype G viral strains downloaded from the Genbank and the sequenced BovHepV contigs (**Supplementary Table 2**). The PCR products were purified with a TIANgel Midi Purification Kit (TIANGEN, China) and then sequenced using the Sanger method.

### 2.5 Genome characterization and phylogenetic analysis

The open reading frames of BovHepV strains were identified using ORFfinder, available on the NCBI website (https://www.ncbi.nlm.nih.gov/orffinder). Sequence identities were evaluated using the MegAlign tool from the DNAstar package version 7.0 and visualized with a heatmap generated in GraphPad Prism 8. Additionally, phylogenetic analysis was performed on the aligned nucleotide and amino acid sequences of BovHepV polyprotein and NS3 protein using the maximum likelihood (ML) method available in MEGA software version 7.0, with a bootstrap value of 1000 replicates (Kumar et al., 2016). A bootstrap value of ≥70 was considered significant and represented in the trees.

### 2.6 Recombination analysis

Recombination events in BovHepV subtype G strains were investigated using seven detection methods provided by the RDP4 package (RDP, GENECONV, Chimaera, MaxChi, BootScan, SiScan, and 3Seq) (Martin et al., 2015). Based on the guidelines provided in the manual, confirmed recombination events were required to fulfill two criteria: i) verification by at least two methods with a *p* value <0.05; ii) an RDP recombination consensus score (RDPRCS) >0.60. If an event satisfied the first criterion but had an RDPRCS between 0.4 and 0.6, it was classified as a potential recombination; otherwise, the event was dismissed.

## 3 Results

### 3.1 Identification of BovHepV

**Figure 1:**
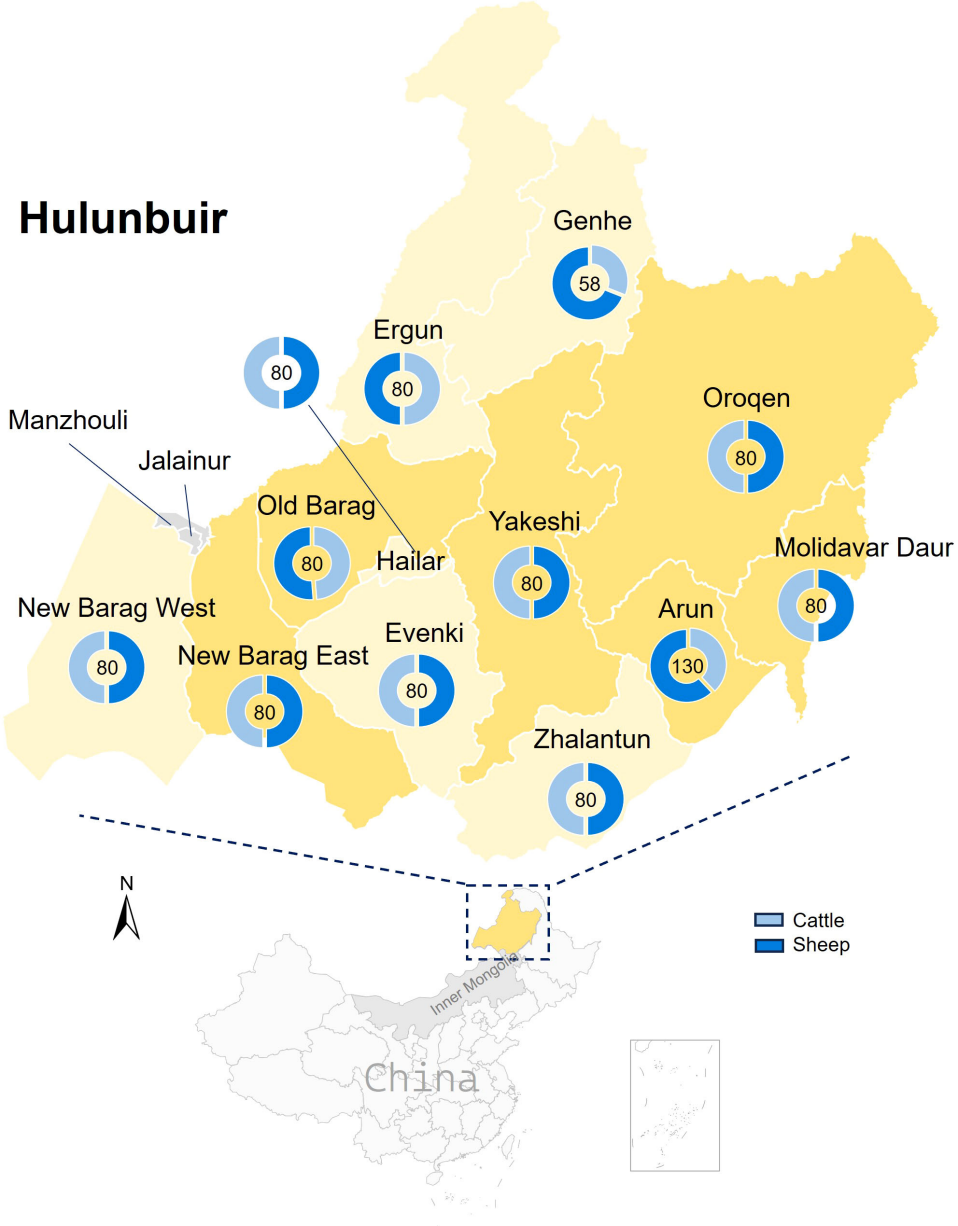
Collection of sheep and cattle serum from Hulunbuir, northeastern China. The yellow regions on the map denote areas in Hulunbuir where bovine hepacivirus has been identified. The blue doughnut chart illustrates the number of samples collected.

A total of 988 blood samples, including 468 samples from cattle and 520 from sheep, were collected and pooled to build 13 RNA libraries (**Figure 1**). Totally, the RNA sequencing resulted in ~13.1 GB of clean data and 15.7 million non-rRNA reads for the cattle serum libraries, as well as ~17.4 GB of clean data and 21.7 million non-rRNA reads for the sheep libraries. From the cattle serum libraries, we identified 37 contigs associated with BovHepV. Notably, one sheep library also revealed two contigs of BovHepV. After being confirmed by semi-nested RT-PCR, a total of 39 serum samples, including 38 from cattle and one from sheep, were identified BovHepV positive (**Table 1**). Among the 12 administrative regions, BovHepV was detected in cattle serum samples from six sites: Old Barag, New Barag East, Oroqen, Arun, Yakeshi, and Molidavar Daur, with prevalence rates ranging from 2.0% to 35.0%. Additionally, one sheep serum sample from Oroqen was tested positive for BovHepV, with a prevalence of 2.5% (**Figure 1**, **Table 1**).

**Table 1:** Prevalence of bovine hepacivirus in sheep and cattle in Hulunbuir, northeastern China.

Region	Cattle		Sheep	
	Positive/size	Prevalence (%)	Positive/size	Prevalence (%)
Old Barag	2/40	5.0	0/40	0.0
New Barag East	5/40	12.5	0/40	0.0
Oroqen	7/40	17.5	1/40	2.5
Evenk	0/40	0.0	0/40	0.0
Genhe	0/18	0.0	0/40	0.0
Arun	1/50	2.0	0/80	0.0
Zhalantun	0/40	0.0	0/40	0.0
Yakeshi	14/40	35.0	0/40	0.0
Ergun	0/40	0.0	0/40	0.0
Morin Dawa	9/40	22.5	0/40	0.0
Hailar	0/40	0.0	0/40	0.0
New Barag West	0/40	0.0	0/40	0.0
Total	38/468	8.1	1/520	0.2

### 3.2 Viral genome characterization and sequence identity

**Figure 2:**
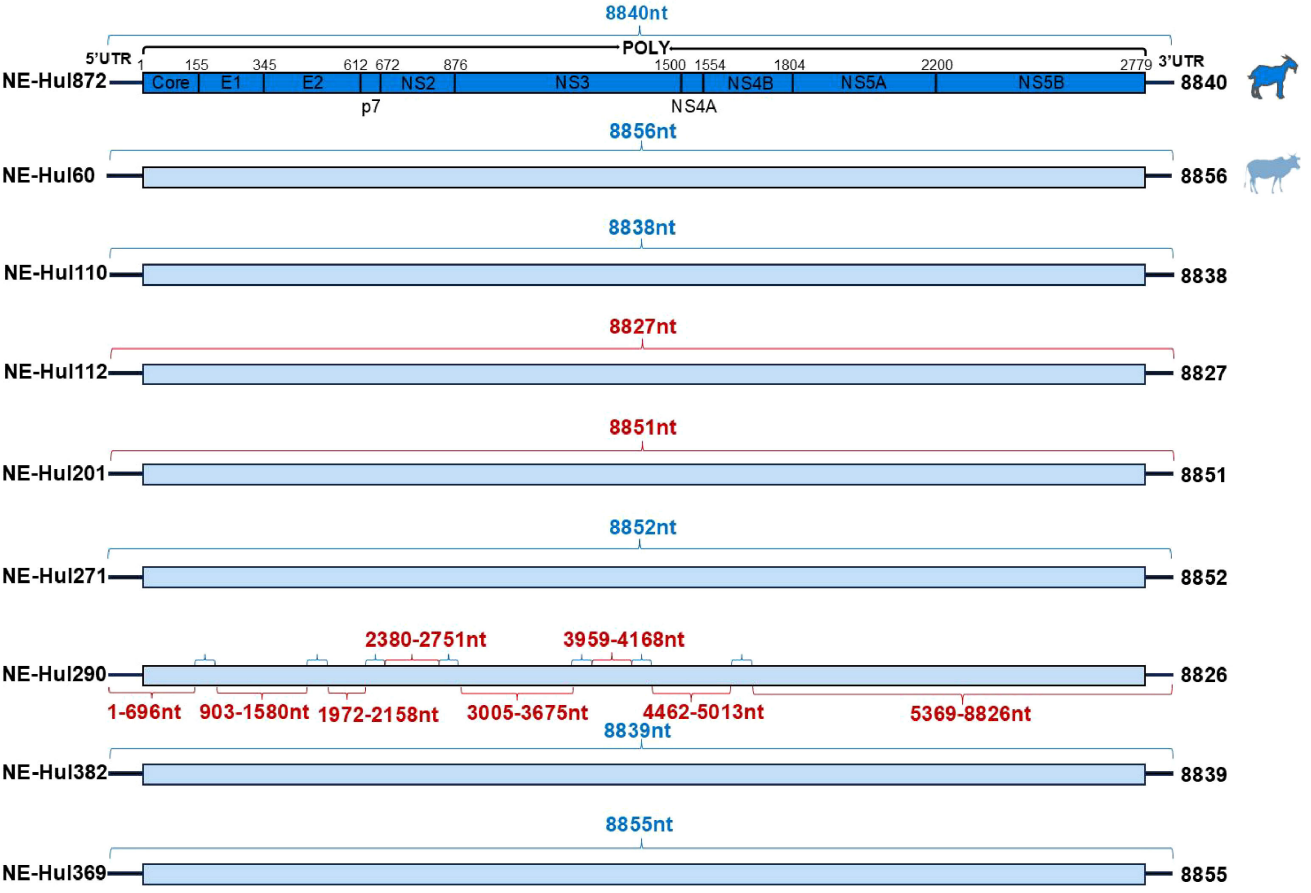
Genomic characterization of bovine hepacivirus (BovHepV) obtained from serum samples of sheep and cattle. Strains obtained from sheep and cattle are represented by deep blue and light blue rectangular boxes, respectively. The sequences of BovHepV derived from metagenomic sequencing are indicated by the blue brace, while those obtained through semi-nested PCR are represented by the red brace.

A total of nine nearly complete genome sequences of BovHepV were amplified and validated by Sanger sequencing, with GenBank accession numbers ranging from PQ304360 to PQ304368 (**Supplementary Table 3**). Among these, eight were derived from cattle and one from sheep. All the nine BovHepV strains possess a large Open Reading Frame (ORF) measuring 8340 nucleotides (nt) in length, capable of encoding a polyprotein comprising 2779 amino acids (aa) (**Figure 2**). This polyprotein is then processed by viral and cellular proteases into the Core protein, two envelope proteins (E1 and E2), along with seven nonstructural proteins (p7, NS2, NS3, NS4A, NS4B, NS5A, and NS5B) (**Figure 2**).

**Figure 3:**
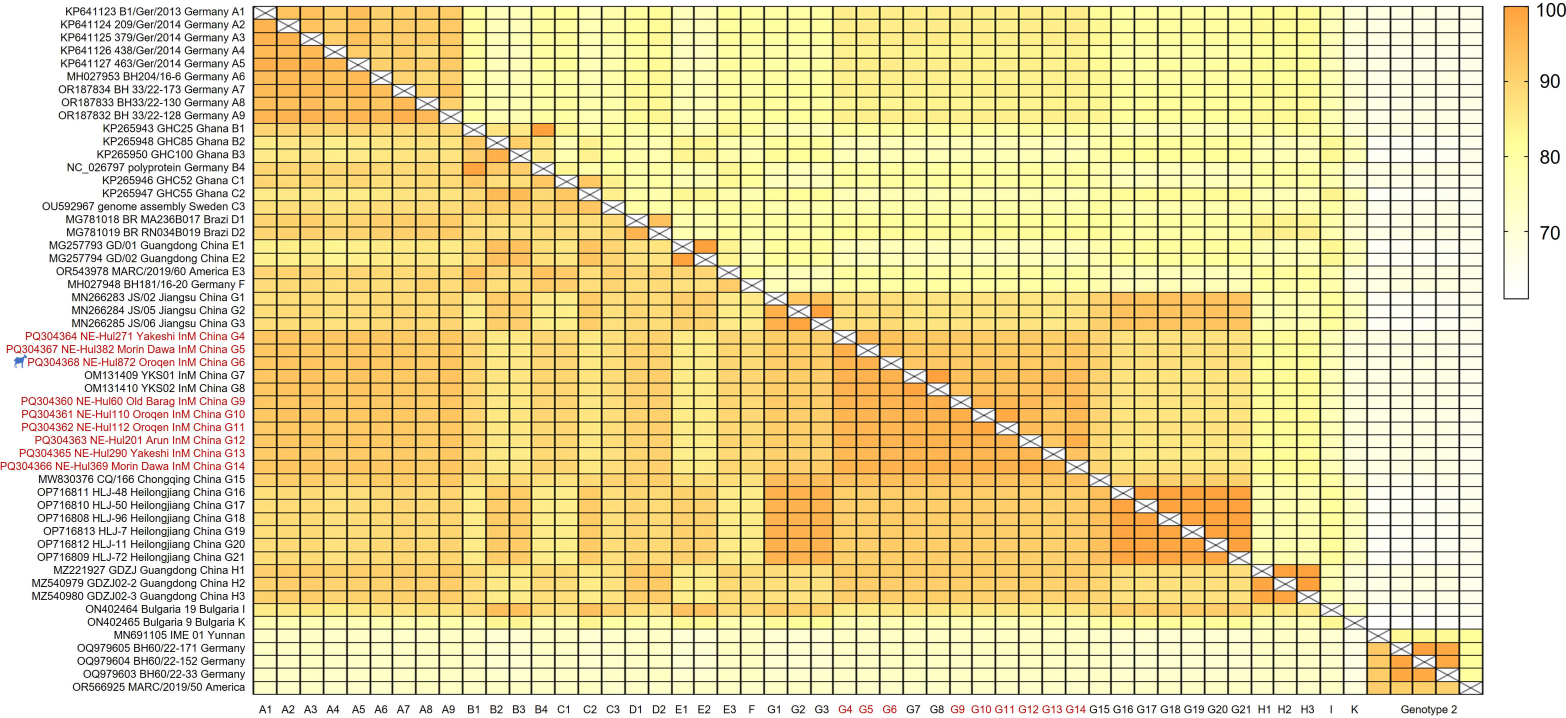
Sequence identities of the bovine hepacivirus polyprotein at both nucleotide (upper right) and amino acid (lower left) levels. The strains identified in this study are denoted by the red font. The strain represented by the silhouette originates from sheep. InM, Inner Mongolia.

Similarity analyses revealed amino acid and nucleotide sequence identities ranging from 95.0% to 98.4% and 90.9% to 97.0% among the nine viral strains, respectively (**Figure 3**, Extended **Table 1**). Notably, the sheep-origin strain showed 91.3–93.8% nucleotide sequence identity and 95.4–96.7% amino acid sequence identity compared to cattle strains. All nine strains demonstrated a close relationship with previously identified BovHepV strains of subtype G from Jiangsu, Chongqing, Heilongjiang, and Inner Mongolia in China, exhibiting over 90% sequence identity at both the amino acid and nucleotide levels (**Figure 3**, Extended **Table 1**).

### 3.3 Phylogenetic analyses

**Figure 4:**
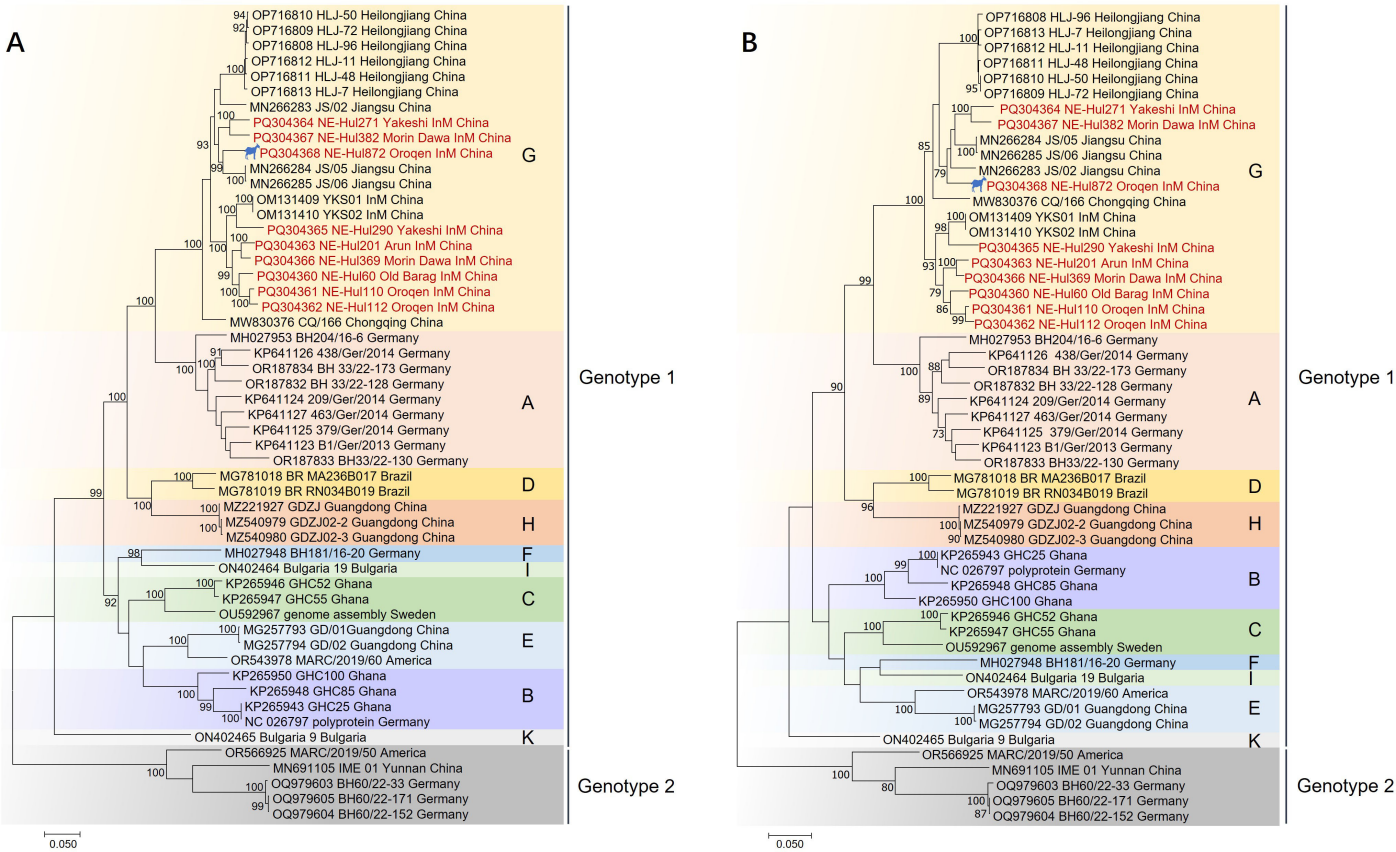
Phylogenetic tree of bovine hepacivirus (BovHepV) based on the nucleotide sequences of polyprotein **(A)** and NS3 protein **(B)**. A bootstrapping analysis comprising 1,000 replicates was performed, with bootstrap values >70 showed in the trees. The strains marked in red represent BovHepV identified in this study. Subtype J strains were excluded due to incomplete genome sequences. The strain denoted by a silhouette is of sheep origin. InM, Inner Mongolia.

Phylogenetic analyses indicated that the BovHepV strains classified under genotype 1 were distinctly segregated into 10 branches, designated as Subtypes A through K (**Figure 4**, **Supplementary Figure 1**). The newly identified strains were found to correspond with subtype G isolates, specifically NE-Hul271, NE-Hul382, and NE-Hul872 (sheep origin), which clustered with strains previously detected in Jiangsu Province. In contrast, the remaining strains exhibited clustering patterns consistent with those observed in Inner Mongolia as reported in our prior study (**Figure 4**, **Supplementary Figure 1**) (Liu et al., 2022a).

### 3.4 Recombination analysis

**Figure 5:**
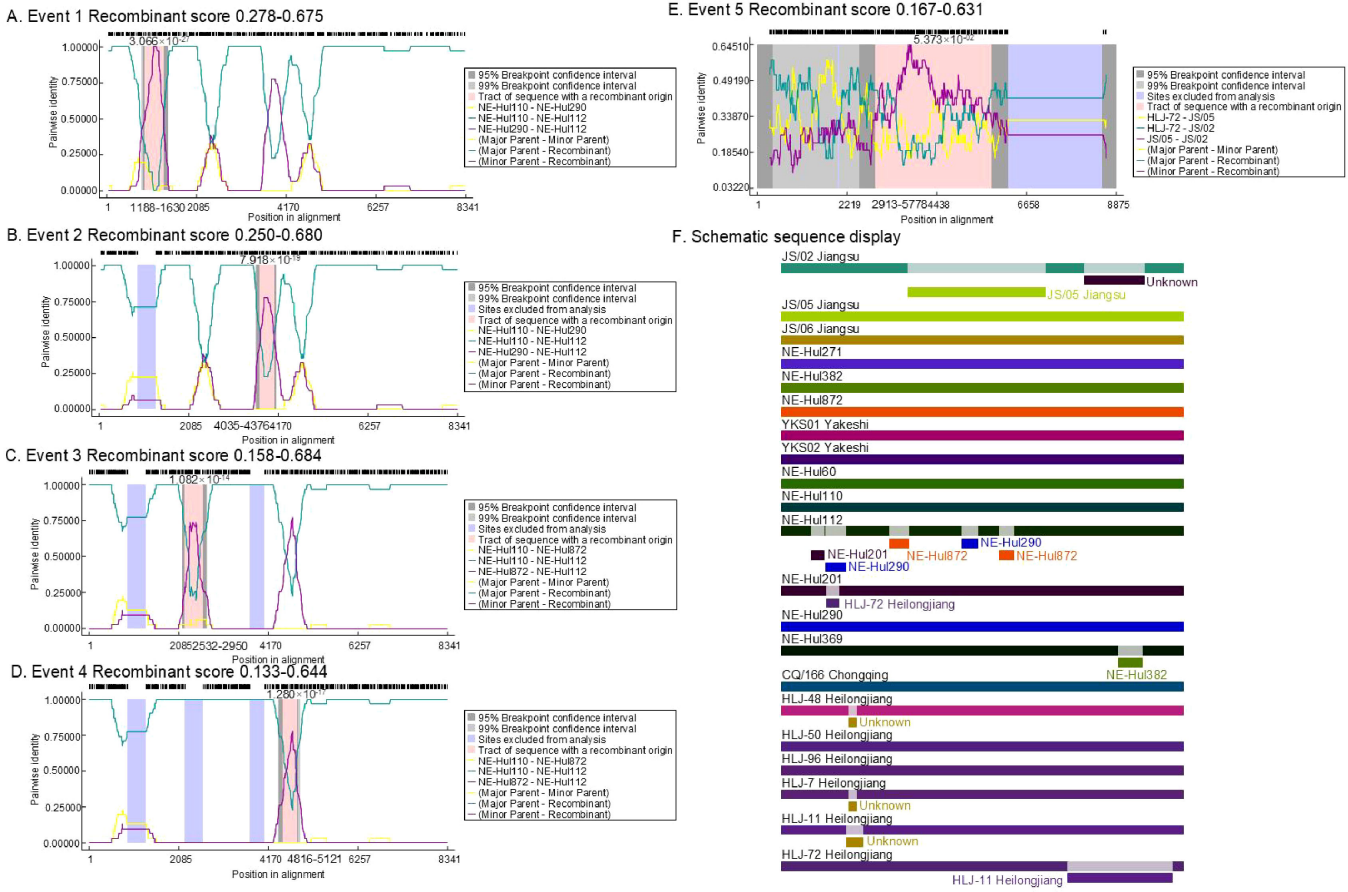
The location and score of confirmed recombinant events among bovine hepacivirus (BovHepV) subtype G strains. **(A–E)**, the location of the recombinant sequences in the five confirmed events; **(F)**, Schematic sequence display of the recombinant sequences among the BovHepV subtype G strains. The schematic representation illustrates predicted recombination events among BovHepV subtype G strains analyzed using the RDP package. Continuous lines indicate no recombination, while likely breakpoint positions are shown below the genome.

The analysis identified a total of 12 predicted recombination events, comprising five confirmed and seven putative events (**Table 2**). Among the five confirmed events, four were associated with strain NE-Hul112 identified in Oroqen, which is predicted to have recombined from the major parent strain NE-Hul110 within the same cattle group and a minor parent strain NE-Hul290 from Yakeshi or strain NE-Hul872 of sheep origin also from Oroqen, indicating the circulation of BovHepV between cattle and sheep (**Figures 5A–F**, **Table 2**). Importantly, a notable event suggests that the sheep-derived strain NE-Hul872 may be involved in the recombination of strain HLJ-7, previously identified in *Rhipicephalus microplus* ticks from cattle in Heilongjiang Province (**Table 2**) (Yuan et al., 2023). Furthermore, some events have also illuminated the long-distance associations among Heilongjiang, Jiangsu, and Inner Mongolia (**Figures 5E, F**, **Table 2**).

**Table 2:** Recombination events detected among bovine hepacivirus subtype G strains using RDP4 package.

Event	Recombinant segment	Major parent	Minor parent	RDPRCS	Tools
1	NE-Hul112	NE-Hul110	NE-Hul290	0.675	RDP, GENECONV, Bootscan, MaxChi, Chimaera, Siscan, 3Seq
2	NE-Hul112	NE-Hul110	NE-Hul290	0.68	RDP, GENECONV, Bootscan, MaxChi, Chimaera, Siscan, 3Seq
3	NE-Hul112	NE-Hul110	NE-Hul290	0.684	RDP, GENECONV, Bootscan, MaxChi, Chimaera, Siscan, 3Seq
4	NE-Hul112	NE-Hul110	NE-Hul872	0.644	RDP, GENECONV, Bootscan, MaxChi, Chimaera, 3Seq
15	JS-02	HLJ-72	JS-05	0.631	MaxChi, Chimaera, Siscan, 3Seq
5	HLJ-7	HLJ-50	NE-Hul872	0.564	RDP, GENECONV, Bootscan, MaxChi, 3Seq
6	NE-Hul112	NE-Hul110	NE-Hul201	0.566	GENECONV, 3Seq
8	HLJ-72	HLJ-50	HLJ-11	0.595	MaxChi,Siscan, 3Seq
9	NE-Hul201	NE-Hul110	HLJ-72	0.511	RDP, MaxChi
11	JS-02	HLJ-48	Unknown	0.447	RDP, Bootscan
12	NE-Hul369	NE-Hul201	NE-Hul382	0.504	RDP, GENECONV, Bootscan, MaxChi, Chimaera, Siscan, 3Seq

## 4 Discussion

As a newly identified hepacivirus, BovHepV has been recognized for nearly a decade, and its widespread distribution and extensive genetic diversity have been confirmed on a global scale (Baechlein et al., 2015; Corman et al., 2015; Canal et al., 2017; Sadeghi et al., 2017; Lu et al., 2018; Yeşilbağ et al., 2018; Lu et al., 2019; Elia et al., 2020; Breitfeld et al., 2022). In China, both genotypes of BovHepV have been identified, with at least three subtypes classified under genotype 1 (Lu et al., 2018; Qiang et al., 2020). Notably, viral strains classified as subtype G appear to have a broader geographical distribution and have been confirmed in Jiangsu, Chongqing, Heilongjiang, and Inner Mongolia (Lu et al., 2019; Liu et al., 2022a; Lu et al., 2022; Yuan et al., 2023). In this study, all nine identified BovHepV strains were classified as subtype G, which further corroborated that subtype G is the predominant strain in northeastern China.

To date, the transmission routes of BovHepV remain incompletely understood. However, the variability in viral prevalence among cattle at different collection sites and the extensive distribution of the virus in Hulunbuir suggest potential rapid transmission pathways for BovHepV, possibly through feces, urine, or nasal discharge (**Table 1**, **Figure 1**). Furthermore, it is reasonable to speculate that vertical transmission may play a role in the epidemiology of BovHepV, as the viral RNA has been detected in commercial fetal bovine serum in previous study (Sadeghi et al., 2017). It is essential to emphasize that cattle are frequently traded across provinces in China, which could promote the transmission of BovHepV and the geographical dissemination of the virus. This is further supported by our recombination analysis, which revealed long-distance associations among BovHepV strains of subtype G from Heilongjiang, Jiangsu, and Inner Mongolia (**Table 2**).

Ticks are likely to play an important role in the transmission of BovHepV, as the virus has been identified in blood-sucking ticks collected from cattle in both Heilongjiang and Guangdong provinces of China (Shao et al., 2021; Yuan et al., 2023). However, to date, no BovHepV RNA has been detected in questing ticks (Liu et al., 2022a, Liu et al., 2022b), which makes it difficult to determine whether the virus was acquired through feeding on cattle blood or if ticks themselves serve as vector hosts for the virus. Therefore, further research is needed to assess the prevalence of BovHepV in questing ticks and their vector competence for the virus.

Hulunbuir, situated in Inner Mongolia, northeastern China, is distinguished for its extensive livestock farming, attributed to the region’s abundant grassland resources (Li et al., 2017). In this region, the high density of cattle and sheep farms is characterized by the shared use of pastures. Consequently, indirect contact with food and feces may significantly enhance intra-and inter-species pathogen transmission. Remarkably, we identified only one positive sample among the 520 sheep, suggesting an incidental spillover event of BovHepV from cattle to sheep. Furthermore, recombination analysis elucidated the parental relationship between the sheep-derived viral strain NE-Hul872 and the cattle-derived strain NE-Hul112 from the same collection site (Oroqen), implying either continuous viral circulation in that region or a recent occurrence of interspecies transmission between sheep and cattle.

Evidence shows that HCV-like viruses can cross species barriers, but cross-species infections involving BovHepV remain exceedingly rare (El-Attar et al., 2015). To date, only two studies have confirmed that BovHepV can infect species other than bovine. One study found subtype F of genotype 1 BovHepV in wild boars in Italy, while another identified genotype 2 BovHepV in a red deer from the Czech Republic (Breitfeld et al., 2022; de Martinis et al., 2022). In the present study, we provide evidence of BovHepV detection in sheep, indicating the potential for this virus to infect additional animal species and thereby broadening the host spectrum of the virus. Although viremia was detected in only one sheep, this suggests that BovHepV may face significant challenges in completing cross-host transmission. Additionally, the short duration of viremia in sheep likely contributes to the low detection rate of BovHepV.

The immune response of the host represents a significant limiting factor for viruses attempting to switch hosts. Research has demonstrated that hepaciviruses can evade the innate immune response by cleaving human adaptor proteins through their proteases (Li et al., 2005; Parera et al., 2012; Anggakusuma et al., 2016). Given that mutations at key amino acid sites may facilitate cross-species transmission, we compared the amino acid differences between the sheep-origin strain and other bovine-origin strains within subtype G. Remarkably, a total of 13 distinct amino acid sites were identified across the E1, E2, NS2, NS3, and NS5B protein regions (**Table 3**), which may represent a key factor facilitating the interspecies transmission of BovHepV from cattle to sheep. This research did not include serological testing for BovHepV, which could lead to an underestimation of the viral prevalence. Considering that acutely infected cattle typically exhibit a clear antibody response followed by subsequent viral clearance (Baechlein et al., 2019; Breitfeld et al., 2022), a combination of molecular biological and serological detection may be suitable for the epidemiological monitoring of the virus.

**Table 3:** Differences in amino acid sites between sheep and cattle derived bovine hepacivirus subtype G strains.

Virus	Mutation sites												
	E1		E2			NS2				NS3		NS5B	
	196	259	360	366	447	722	771	778	835	1229	1362	2442	2586
NE-Hul872^#^	S	S	T	Y	N	F	S	R	F	I	P	R	I
JS/02	N	H	A	H	K	L	G	K	Y	L	S	K	V
JS/05	D	R	A	H	K	L	G	K	Y	L	S	K	V
JS/06	D	R	A	H	K	L	G	K	Y	L	S	K	V
NE-Hul271	D	H	A	H	Q	L	G	K	Y	L	S	K	V
NE-Hul382	D	H	A	H	K	L	G	K	Y	L	S	K	V
YKS01	D	H	A	H	Q	L	G	K	Y	L	S	K	V
YKS02	D	H	A	H	Q	L	G	K	Y	L	S	K	V
NE-Hul60	D	H	A	H	K	L	G	K	Y	L	S	K	V
NE-Hul110	D	H	A	H	Q	L	G	K	Y	L	S	K	V
NE-Hul112	D	H	A	H	Q	L	G	K	Y	L	S	K	V
NE-Hul201	N	H	A	H	E	L	G	K	Y	L	S	K	V
NE-Hul290	D	H	A	H	K	L	G	K	Y	L	S	K	V
NE-Hul369	D	H	A	H	Q	L	G	K	Y	L	S	K	V
CQ/166	D	H	A	H	Q	L	G	K	Y	L	S	K	V
HLJ-48	D	H	V	H	K	L	G	K	Y	L	S	K	V
HLJ-50	D	H	V	H	K	L	G	K	Y	L	S	K	V
HLJ-96	D	H	V	H	K	L	G	K	Y	L	S	K	V
HLJ-7	D	H	V	H	K	L	G	K	Y	L	S	K	V
HLJ-11	D	H	V	H	K	L	G	K	Y	L	S	K	V
HLJ-72	D	H	V	H	K	L	G	K	Y	L	S	K	V

^#^The strain marked in red is originally derived from sheep.

In conclusion, we identified genetically diverse BovHepV strains of subtype G within genotype 1 in cattle from Hulunbuir, northeastern China. Furthermore, an additional BovHepV strain in sheep were detected for the first time, confirming cross-species transmission and co-circulation of the virus between cattle and sheep, thereby expanding the host range of the virus.

## Data Availability

The datasets presented in this study can be found in online repositories. The names of the repository/repositories and accession number(s) can be found below: https://www.ncbi.nlm.nih.gov/genbank/, PQ304360 https://www.ncbi.nlm.nih.gov/genbank/, PQ304361 https://www.ncbi.nlm.nih.gov/genbank/, PQ304362 https://www.ncbi.nlm.nih.gov/genbank/, PQ304363 https://www.ncbi.nlm.nih.gov/genbank/, PQ304364 https://www.ncbi.nlm.nih.gov/genbank/, PQ304365 https://www.ncbi.nlm.nih.gov/genbank/, PQ304366 https://www.ncbi.nlm.nih.gov/genbank/, PQ304367 https://www.ncbi.nlm.nih.gov/genbank/, PQ304368 https://www.ncbi.nlm.nih.gov/, PRJNA1164459.
