# Supplementary material for: Detection and characterization of bovine hepacivirus in cattle and sheep from Hulunbuir, northeastern China
Source: Front Cell Infect Microbiol. 2025 Jan 28;15:1540849. doi: 10.3389/fcimb.2025.1540849 (PMC11811627; doi:10.3389/fcimb.2025.1540849)
Supplement: Supplementary file 2 [file DataSheet2.docx]

Supplementary Material

**Supplementary Table 1.** Primers used for the identification of bovine hepacivirus.

| **Primers** | **Sequence (5′ → 3′)** | **Amplicon (bp)** |
| --- | --- | --- |
| BovHepV-1702F ^a^ | TCACGTCTTACCACRCCGGAA | 515 |
| BovHepV-2217R2 ^b^ | AAAGCGATAAGGTCTACGACT |  |
| BovHepV-2363R1 ^b^ | GTCGCAAACARYCCRGAACCA |  |
|  |  |  |
| BovHepV-1374F1 ^a^ | CAAATGGGGACATCTAACCAGT | 539 |
| BovHepV-1396F2 ^a^ | TGCTCCCTTCAAATGATACCCT |  |
| BovHepV-1935R ^b^ | ACACTAGACCAGTAGCGAGA |  |
|  |  |  |
| BovHepV-1332F ^a^ | GCGAATTGGTACTGTTATCCCT | 250 |
| BovHepV-1582R2 ^b^ | TCCCAACTAGTGCCAATCGT |  |
| BovHepV-1611R1 ^b^ | AACCCGTTGGTGCATAGACA |  |
|  |  |  |
| BovHepV-670F ^a^ | ATTTCGTGAACGGTCTGACT | 428 |
| BovHepV-1098R2 ^b^ | TGCCAAAAGGGGAATAATCCAC |  |
| BovHepV-1030R1 ^b^ | ATTCACAGCCCAATTAACCCAC |  |
|  |  |  |
| BovHepV-1178F ^a^ | GTCAATAGTTACCCGCATACCTT | 421 |
| BovHepV-1599R2 ^b^ | GCAAATAGGATCACGGTCCAT |  |
| BovHepV-1637R1 ^b^ | GTCTTTCCATGTGCAATCCGTA |  |

*a, Forward; b, Reverse.

**Supplementary Table 2.** Primers used for the genome amplification of bovine hepacivirus.

| **Primers** | **Sequence (5′ → 3 ′)** | **Amplicon (bp)** |
| --- | --- | --- |
| BovHepV-15F ^a^ | ATTAAGTTAGGTTCCGCCGAAG | 705 |
| BovHepV-720R2 ^b^ | YRCYACGCTACAAAGACACAA |  |
| BovHepV-750R1 ^b^ | CCCACSTYRACCWGTCGGTA |  |
|  |  |  |
| BovHepV-587F1 ^a^ | CRTDTGYCGCAAGDTYCCAA | 349 |
| BovHepV-906F2 ^a^ | GTYARGCCNAATGGTTCCACA |  |
| BovHepV-1255R ^b^ | GCCCYTGAAYYGCAAACCACA |  |
|  |  |  |
| BovHepV-906F ^a^ | GTYARGCCNAATGGYTCCACA | 522 |
| BovHepV-1428R2 ^b^ | ATCCTCAACAKMGTARGTCAC |  |
| BovHepV-1464R1 ^b^ | GGGRACATTMACCACRGCRTC |  |
|  |  |  |
| BovHepV-1105F1 ^a^ | AYTCYCCYTTTGGYACTATYGTT | 562 |
| BovHepV-1180F2 ^a^ | CRATAGTYACHCGCATACCTT |  |
| BovHepV-1742R ^b^ | ACATRGCRGCCCGAACNATCCAR |  |
|  |  |  |
| BovHepV-1599F1 ^a^ | RYGGACCGTGAYCCTATTTGC | 513 |
| BovHepV-1642F2 ^a^ | ATTGYACATGGAAASABCCY |  |
| BovHepV-2155R ^b^ | AATCRTCCCAGGTBGAGGCTA |  |
|  |  |  |
| BovHepV-1892F1 ^a^ | GGTYTTYTCYCCTARYGGAAC | 594 |
| BovHepV-1920F2 ^a^ | CCTYTNGGHGATCTCGCYAC |  |
| BovHepV-2514R ^b^ | KATRGCDAGTAACCCCATRCT |  |
|  |  |  |
| BovHepV-2317F1 ^a^ | AAACTYAYRCTCCTTTAGCCT | 634 |
| BovHepV-2344F2 ^a^ | CTTTCCTARCTCTYGTHGGTT |  |
| BovHepV-2978R ^b^ | CCAAAGHGCCGTRTCAATTCCC |  |
|  |  |  |
| BovHepV-2875F1 ^a^ | AAACTYAYRCTCCTTTAGCYT | 607 |
| BovHepV-2908F2 ^a^ | CATGYTCRCCTATCGGYGTY |  |
| BovHepV-3515R ^b^ | ANCCVGTTGGTGCATAGACA |  |
|  |  |  |
| BovHepV-3413F1 ^a^ | CTGYACCACNCCAGTNAGACAC | 574 |
| BovHepV-3470F2 ^a^ | TCTCTCRGCACCRCCRACGR |  |
| BovHepV-4044R ^b^ | CCCCTAGACCGRAGRTCAGC |  |
|  |  |  |
| BovHepV-3865F ^a^ | TGCTTGCYACTGCYACBCC | 498 |
| BovHepV-4363R2 ^b^ | ACACRGAWGCCTCAGAAACRG |  |
| BovHepV-4391R1 ^b^ | CAHGCCATACCRCTGTCRAAC |  |
|  |  |  |
| BovHepV-4260F ^a^ | RTACCAGCYAAYTCAGTCACDC | 581 |
| BovHepV-4841R2 ^b^ | GCCACYGAYCCAAAGTAGTCR |  |
| BovHepV-4930R1 ^b^ | CAGCACTCYTCRAASCCRT |  |
|  |  |  |
| BovHepV-4731F1 ^a^ | CCHGTGCCTGAAATTGTNAAYC | 563 |
| BovHepV-4810F2 ^a^ | CGGCCTCNGTYTTYGCYCTYG |  |
| BovHepV-5373R ^b^ | CRAGYAGCTTGAAAACAACTCC |  |
|  |  |  |
| BovHepV-5113F1 ^a^ | CYGGYATACTGATATCNYCT | 596 |
| BovHepV-5171F2 ^a^ | YGCCGTRACACTCCCRCTA |  |
| BovHepV-5767R ^b^ | ATVCCYCCGAYGAGAGCTT |  |
|  |  |  |
| BovHepV-5529F ^a^ | AAYAGGCTCTTGTCYATGGTT | 527 |
| BovHepV-6056R2 ^b^ | CCATMCGRTACTCCACRAAHCCR |  |
| BovHepV-6139R1 ^b^ | CCGCRCTGAAYACCCTAGGAA |  |
|  |  |  |
| BovHepV-5801F1 ^a^ | CCTYCCYGGBGTYCCYGTA | 558 |
| BovHepV-5975F2 ^a^ | CGTRCCYGTNAAYAACACC |  |
| BovHepV-6533R ^b^ | CCGCCCCARAAGTARGACTCA |  |
|  |  |  |
| BovHepV-6345F1 ^a^ | GACCCYTTYACYACYGACGTT | 719 |
| BovHepV-6387F2 ^a^ | GCTGTYTCTAATGARCGYGCTGA |  |
| BovHepV-7106R ^b^ | GCTGCATGTCCAATGTTGARACC |  |
|  |  |  |
| BovHepV-6959F1 ^a^ | CATCACCCAYGGAYTGGTBT | 523 |
| BovHepV-7043F2 ^a^ | CTTYAGRCCTCCRBCTACACC |  |
| BovHepV-7566R ^b^ | HGCTTCRCGRTCRACATCCTC |  |
|  |  |  |
| BovHepV-7509F ^a^ | CCYAAYCCYATGGCATTYG | 545 |
| BovHepV-8054R2 ^b^ | CYTCACCYTCHGCNGAGCAR |  |
| BovHepV-8074R1^b^ | GGCRCCCATYGGRTTRAACC |  |
|  |  |  |
| BovHepV-8019F ^a^ | TTCTTGACRCGCGAYCCTAGY | 507 |
| BovHepV-8526R2 ^b^ | CCCGATBACAATGGGTTCCTC |  |
| BovHepV-8580R1 ^b^ | GYAAAGCCACAAYAGATAAAGC |  |
|  |  |  |
| BovHepV-8035F1 ^a^ | CTAGYGTTCCYTTTGCRCGYT | 740 |
| BovHepV-8075F2 ^a^ | GTTYAAYCCRATGGGYGCCT |  |
| BovHepV-8815R ^b^ | ACCAACCTCAAAGCGGGTAG |  |

*a Forward; b Reverse.

**Supplementary Table 3.** The detailed information of the amplified nine strains of bovine hepacivirus.

| **Accession number** | **Strain** | **Country** | **Host** |
| --- | --- | --- | --- |
| PQ304360 | NE-Hul60 | China: Old Barag, Inner Mongolia | cattle |
| PQ304361 | NE-Hul110 | China: Oroqen, Inner Mongolia | cattle |
| PQ304362 | NE-Hul112 | China: Oroqen, Inner Mongolia | cattle |
| PQ304363 | NE-Hul201 | China: Arun, Inner Mongolia | cattle |
| PQ304364 | NE-Hul271 | China: Yakeshi, Inner Mongolia | cattle |
| PQ304365 | NE-Hul290 | China: Yakeshi, Inner Mongolia | cattle |
| PQ304366 | NE-Hul369 | China: Molidavar Daur, Inner Mongolia | cattle |
| PQ304367 | NE-Hul382 | China: Molidavar Daur, Inner Mongolia | cattle |
| PQ304368 | NE-Hul872 | China: Oroqen, Inner Mongolia | sheep |


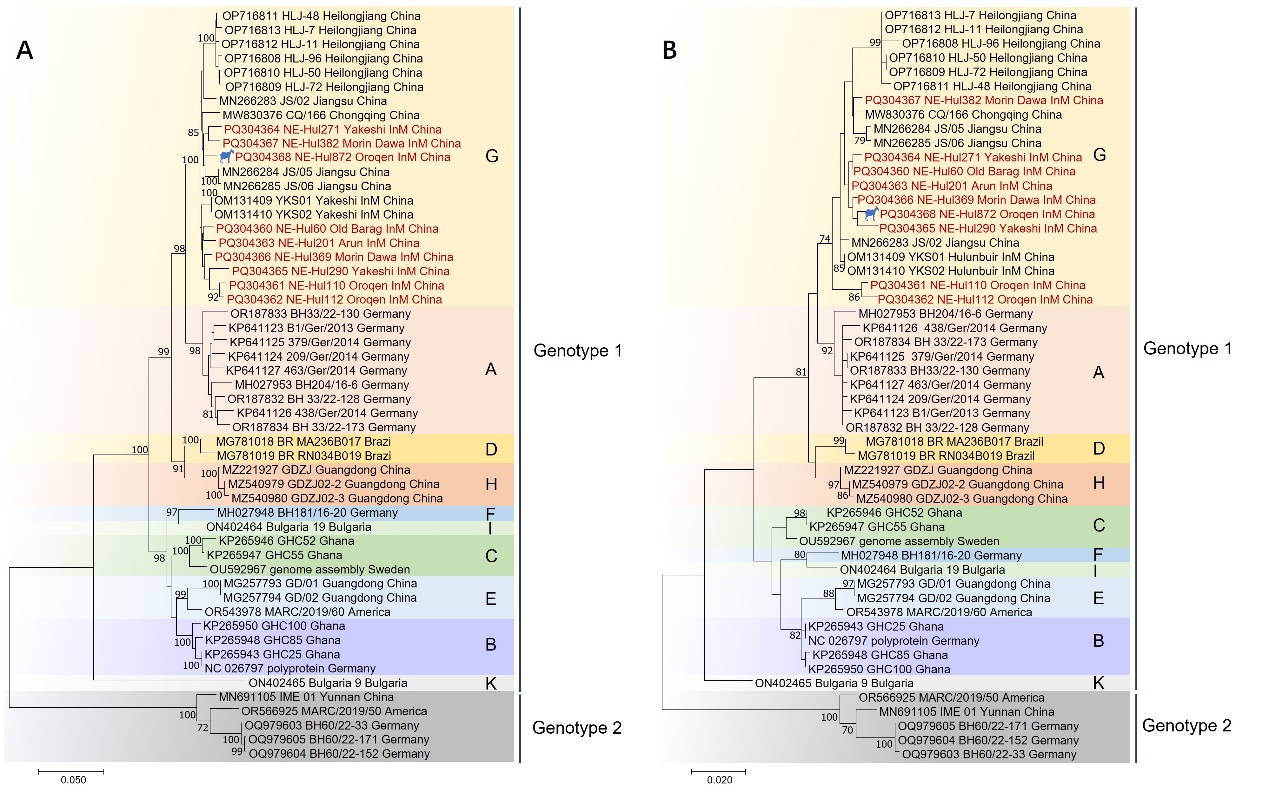


**Supplementary Figure 1.** Phylogenetic tree of bovine hepacivirus (BovHepV) based on the amino acid sequences of polyprotein (A) and NS3 protein (B). A bootstrapping analysis comprising 1,000 replicates was performed, with bootstrap values >70 showed in the trees. The strains marked in red represent BovHepV identified in this study. Subtype J strains were excluded due to incomplete genome sequences. The strain denoted by a silhouette is of sheep origin. InM, Inner Mongolia.
